# Choanoflagellates and the ancestry of neurosecretory vesicles

**DOI:** 10.1098/rstb.2019.0759

**Published:** 2021-03-29

**Authors:** Ronja Göhde, Benjamin Naumann, Davis Laundon, Cordelia Imig, Kent McDonald, Benjamin H. Cooper, Frédérique Varoqueaux, Dirk Fasshauer, Pawel Burkhardt

**Affiliations:** ^1^ Sars International Centre for Molecular Marine Biology, University of Bergen, 5006 Bergen, Norway; ^2^ Institute of Zoology and Evolutionary Research, Friedrich Schiller University Jena, 07743 Jena, Germany; ^3^ Marine Biological Association of the United Kingdom, The Laboratory, Citadel Hill, Plymouth PL1 2PB, UK; ^4^ Department of Molecular Neurobiology, Max Planck Institute of Experimental Medicine, 37075 Gottingen, Germany; ^5^ Electron Microscope Laboratory, University of California, Berkeley, CA 94720, USA; ^6^ Department of Fundamental Neurosciences, University of Lausanne, 1005 Lausanne, Switzerland

**Keywords:** choanoflagellate, vesicle, cell polarity, SNARE, evolution, synaptobrevin

## Abstract

Neurosecretory vesicles are highly specialized trafficking organelles that store neurotransmitters that are released at presynaptic nerve endings and are, therefore, important for animal cell–cell signalling. Despite considerable anatomical and functional diversity of neurons in animals, the protein composition of neurosecretory vesicles in bilaterians appears to be similar. This similarity points towards a common evolutionary origin. Moreover, many putative homologues of key neurosecretory vesicle proteins predate the origin of the first neurons, and some even the origin of the first animals. However, little is known about the molecular toolkit of these vesicles in non-bilaterian animals and their closest unicellular relatives, making inferences about the evolutionary origin of neurosecretory vesicles extremely difficult. By comparing 28 proteins of the core neurosecretory vesicle proteome in 13 different species, we demonstrate that most of the proteins are present in unicellular organisms. Surprisingly, we find that the vesicular membrane-associated soluble N-ethylmaleimide-sensitive factor attachment protein receptor protein synaptobrevin is localized to the vesicle-rich apical and basal pole in the choanoflagellate *Salpingoeca rosetta.* Our 3D vesicle reconstructions reveal that the choanoflagellates *S. rosetta* and *Monosiga brevicollis* exhibit a polarized and diverse vesicular landscape reminiscent of the polarized organization of chemical synapses that secrete the content of neurosecretory vesicles into the synaptic cleft. This study sheds light on the ancestral molecular machinery of neurosecretory vesicles and provides a framework to understand the origin and evolution of secretory cells, synapses and neurons.

This article is part of the theme issue ‘Basal cognition: multicellularity, neurons and the cognitive lens’.

## 1. Introduction

Coordinated cell–cell signalling is required for an organized and adaptive behaviour of different cells and cell types, which is, in turn, crucial for the evolution of body plans in multicellular organisms. Two major modes of signalling can be found in animals, volume transmission and synaptic signalling. Volume transmission or paracrine signalling is mediated by diffusion of chemical signals and does not require direct cell–cell contact sites. However, volume transmission is limited by diffusion rates and therefore requires either a small body size or a circulatory system distributing signalling molecules through a larger body. Synaptic transmission in contrast relies on the release of electrical or chemical signals at specific cellular membrane contact sites called synapses. Electric synapses allow direct and fast communication by the transport of ions through specific channels called gap junctions, that connect the cellular membranes of the two nerve cells. Chemical synapses release chemical substances (e.g. neuropeptides or neurotransmitters) from the presynaptic site of a signal sending cell to the postsynaptic site of a signal receiving cell over larger distances. Pre- and postsynapse are separated by a small space, the synaptic cleft, over which neurotransmitters diffuse to their target receptors localized on the postsynaptic site of connected cells resulting in signal transmission between different cells (for reviews, see [1,2]). This mode allows an efficient signalling even in larger bodies by the formation of cell extensions and the establishment of synaptic and neuronal circuits. In animals, a variety of highly specialized neuronal cell types has evolved, facilitating synaptic signal transmission over large distances and eventually building up nervous systems. This feature might have been a crucial prerequisite for the evolution of increasing body sizes and the diversification of animal body plans [3]. Moreover, synapses played a pivotal role in the evolution of the cognitive system of animals. Cognition, defined as the biology of information processing [4], is tightly connected to life itself and can, therefore, also be found in single-celled organisms. However, cognitive processes demand fast and reliable mechanisms of signal transduction. While volume transmission-based signal transmission might be sufficient for unicellular and colonial organisms, it becomes insufficient for organisms that have larger, more complex bodies [5]. Hence, synaptic signal transmission might have been crucial to allow for cognitive processes in large multicellular organisms with complex body structures.

Even though animal synapses have been extensively studied through several decades, their evolutionary origin is still unresolved [6–10]. However, reconstructing the evolutionary origin of the first synapses is an important landmark to understand how cognitive processes in single-celled organisms were altered and adapted to function in multicellular organisms.

To elucidate the evolution of synapses in animals, it is necessary to investigate the presence and ancestral function of key synaptic components in closely related unicellular organisms. Neurosecretory vesicles that store neuropeptides or neurotransmitters at the presynaptic site of a nerve cell constitute one of these key components. At least two morphologically distinct types of neurosecretory vesicles are involved in neuropeptide or neurotransmitter secretion of neurons: small synaptic vesicles (SVs) and large dense core vesicles (DCVs) [11,12]. Small SVs store classical neurotransmitters, like for example glutamate or acetylcholine, and have a diameter of 30–40 nm. DCVs have a diameter of 80–200 nm and an electron-dense core filled with neuropeptides (reviewed by [13,14]). Neurosecretory vesicles, concentrated at pre-synapses, facilitate signal propagation via the fusion of the vesicular membrane with the presynaptic plasma membrane and subsequent neurotransmitter release into the synaptic cleft. In endocrine and neuroendocrine cells, but also in neurons, DCVs function in multiple biological processes, for example, the development of the brain [15], synaptic plasticity [16], behaviour [17] or circadian rhythms [18] via the release of proteins or neuropeptides. In neurons, DCVs are found in many different parts of the cell, including dendrites, axonal varicosities and synaptic terminals, where they fulfil important roles for synaptic transmission, memory formation and neuronal survival [19].

**Figure 1.**
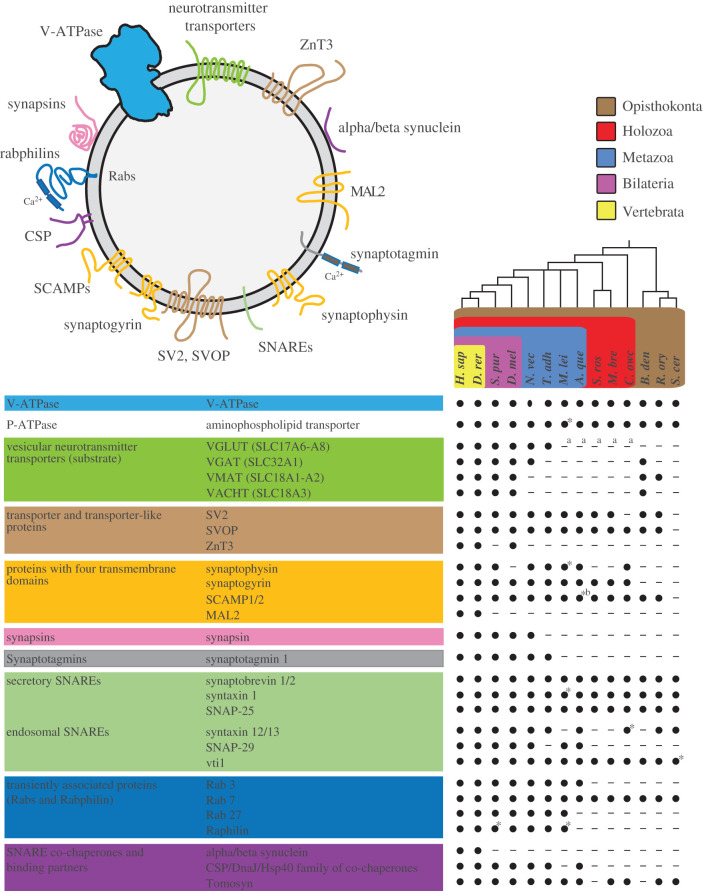
Neurosecretory vesicle proteins in animals and their closest living relatives. (Top) Schematic model of the core molecular components of animal neurosecretory vesicles. (Below) Core proteins of animal neurosecretory vesicles can be assigned to ten categories: V-ATPases, vesicular neurotransmitter transporters, transporter and transporter-like proteins, proteins with four transmembrane domains, synapsins, synaptotagmins, secretory SNAREs, endosomal SNAREs, transiently associated proteins SNARE binding partners and co-chaperones (modified after [24]). Black dots indicate the presence of clear protein sequence homologues (also see electronic supplementary material, table S1), while lines indicate that a homologue was not detected in the respective organism. Taxonomic groupings are indicated as follows: brown box, Opisthokonta; red box, Holozoa; blue box, Metazoa; violet box, Bilateria; yellow box, Vertebrata. Phylogenetic tree based on a consensus phylogeny [25–28]. *A. que*, *Amphimedon queenslandica*; *B. den*, *Batrachochytrium dendrobatidis*; *C. owc*, *Capsaspora owczarzaki*; *D. rer*, *Danio rerio*; *D. mel*, *Drosophila melanogaster*; *H. sap*, *Homo sapiens*; *M. bre*, *Monosiga brevicollis*; *M. lei*, *Mnemiopsis leidyi*; *N. vec, Nematostella vectensis*; *R. ory*, *Rhizopus oryzae*; *S. cer*, *Saccharomyces cerevisae*; *S. pur*, *Strongylocentrotus purpuratus*; *S. ros*, *Salpingoeca rosetta*; *T. adh*, *Trichoplax adhaerens*. *B. den*, *R. ory* and *S. cer* are fungi. * = protein of interest-like, ^a^ = putative SLC17A5-homologue, ^b^ = domain structure lost.

The protein compositions of these two classes of neurosecretory vesicle membrane are well characterized [20–22]. Despite different cargos and biological roles, the protein composition of neurosecretory vesicles appears to be similar [23] and is composed of a set of core proteins, which can be assigned to specific categories (figure 1; [24]). These categories include trafficking proteins, such as synaptotagmins that act as calcium sensors [29]; Rab proteins that play important roles in docking and tethering neurosecretory vesicles to the presynaptic membrane [30]; soluble N-ethylmaleimide-sensitive factor attachment protein receptors (SNAREs), which mediate membrane fusion [31,32]; and proteins that are believed to be SNARE co-chaperones and SNARE binding partners [33–35]. Among these categories are also four transmembrane proteins that possess four membrane-spanning helices; the phosphoprotein family synapsin which are specifically associated on SVs [36]; transmembrane adenosine triphosphatases (ATPases) that use the free energy derived from ATP hydrolysis to transport metabolites across membranes, as well as other transporters and transporter-like proteins (figure 1).

Choanoflagellates are the closest unicellular relatives of animals and exhibit a surprisingly rich repertoire of neuronal protein homologues [37–41]. The recent observations of morphologically distinct intracellular vesicle populations [42] and the presence of plasma membrane contacts between colonial cells in the choanoflagellate *Salpingoeca rosetta* [43] are particularly interesting as they shed light on potential precursors involved in cellular specialization mechanisms in animals. These features—neuronal proteins, plasma membrane contacts and the presence of distinct vesicle populations—are also important components of the synaptic neurosecretory system in animals, emphasizing the benefits of choanoflagellates as a model to investigate the evolutionary origin of animal synapses.

In the present study, we performed a comparative analysis of neurosecretory vesicle proteins together with a morphological characterization of the vesicle types in *S. rosetta* and *Monosiga brevicollis*. Using comparative cross-species protein analysis in combination with immunohistochemistry, serial ultrathin transmission electron microscopy (ssTEM) and 3D reconstruction, we show that choanoflagellates exhibit a rich repertoire of neurosecretory vesicle proteins and a diverse vesicular landscape (distributed along the apical–basal axis of the cell). Based on their morphology and localization, we could assign the vesicles to five different classes, of which some were specifically localized in close proximity to the cell poles. Together with the overlapping immunostaining signals of the cytoskeleton protein tubulin and the secretory vesicle marker synaptobrevin, our findings indicate a directed protein transport in *S. rosetta* towards the periphery of the cell similar to the transport system in many animal polarized cells, including neurons.

## 2. Results

### (a) Comparative analysis reveals the ancestry of neurosecretory vesicle proteins

Neurosecretory vesicles are composed of a ‘core proteome’ that can be subdivided into specific categories: ATPases; transporters and transporter-like proteins; proteins with four transmembrane domains; synapsins; synaptotagmins; SNAREs; SNARE co-chaperones; SNARE binding partners and Rab proteins (figure 1). Based on this core proteome, we selected 28 proteins with at least one representative from each category to perform a survey for respective homologues. This survey was conducted in a total of 13 different eukaryotic species, covering animals that have clearly recognizable neurons (zebrafish (*Danio rerio*), sea urchin (*Strongylocentrotus purpuratus*), fruit fly (*Drosophila melanogaster*), sea anemone (*Nematostella vectensis*), ctenophore (*Mnemiopsis leidyi*)), animals without recognizable neurons (placozoan (*Trichoplax adhaerens*), sponge (*Amphimedon queenslandica*)), their closest unicellular relatives (two choanoflagellate species (*S. rosetta*, *M. brevicollis*), filasterean (*Capsaspora owczarzaki*)) and three fungal species (*Batrachochytrium dendrobatidis*, *Rhizopus oryzae*, *Saccharomyces cerevisiae*) (figure 1). *Batrachochytrium dendrobatidis* belongs to the chytrids, fungi that have flagellated cells and are considered as the sister group of the other non-flagellated fungi [44] (figure 1).

Overall, we found that approximately 39% of the examined neurosecretory vesicle proteins are restricted to animals. The following proteins were only found in animals: synapsin, one of the most abundant SV proteins [20]; the synaptic-associated zinc transporter ZnT3 [45]; the calcium sensor synaptotagmin1 [46]; the co-chaperone cysteine string protein (CSP) [33]; myelin and lymphocyte protein 2 (MAL2) [23]; and synuclein [47]. Strikingly, and in accordance with previous studies [39,48–52], our results show that the majority (approx. 61%) of the examined neurosecretory vesicle proteins are also present in unicellular opisthokonts (figure 1). We found secretory SNAREs, Rab7, V- and P-ATPase protein sequences in all investigated organisms. We also identified the ‘four transmembrane domain protein’ synaptophysin in the unicellular eukaryote *C. owczarzaki*. Synaptogyrin was also found in *C. owczarzaki*, as well as in the two choanoflagellate species *M. brevicollis* and *S. rosetta*. In addition, we found the synaptic vesicle protein 2 (SV2) in most of the investigated organisms, and the SV2-related protein (SVOP) in all species except for *S. cerevisiae*.

The analysed neurotransmitter transporters showed diverse presence and absence patterns for the different species. Vesicular glutamate transporters (VGLUT) were found in all investigated bilaterians, *N. vectensis* and *T. adhaerens*, but vesicular monoamine transporters (VMAT) and vesicular acetylcholine transporters (VAChT) appeared only in bilaterians and in some of the investigated fungi. The vesicular GABA transporter (VGAT) was found in all investigated bilaterians, *N. vectensis* and in the fungus *B. dendrobatidis.* However, this transporter appears to be absent in *T. adhaerens*, *M. leidyi*, *A. queenslandica*, *S. rosetta*, *M. brevicollis* and *C. owczarzaki*.

In short, our comparative analysis revealed that approximately 61% of the core neurosecretory vesicle proteins evolved before the emergence of the first animals. To further assess the evolutionary origin of neurosecretory vesicles, we used the vesicle-associated protein synaptobrevin as marker for the presence and localization of putative secretory vesicles in the choanoflagellate *S. rosetta*. The life history of *S. rosetta* involves several sexual and asexual unicellular and multicellular stages [53,54]. *Salpingoeca rosetta* is, therefore, a suitable model to investigate the evolutionary origin of neurosecretory vesicle-based signalling between cells in animals. *M. brevicollis*, in contrast, is a choanoflagellate that occurs only in single cell stages. Additionally, both species are the only two choanoflagellates for which a complete genome is available [25,37]. A comparison of these both closely related, but very different species might help to reconstruct the evolution of neurosecretory vesicle-based cell signalling in animals.

### (b) Synaptobrevin as a putative secretory vesicle marker in the choanoflagellate *S. rosetta*

**Figure 2.**
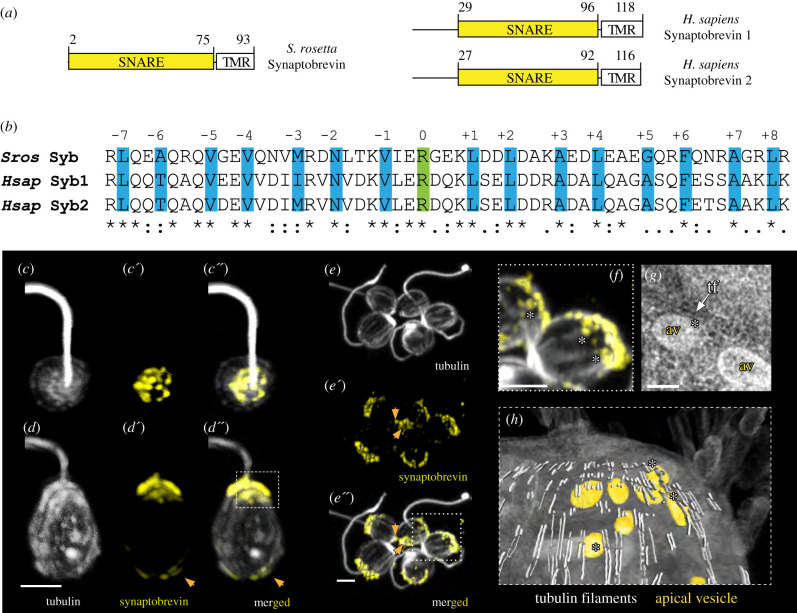
Synaptobrevin in the choanoflagellate *Salpingoeca rosetta*. (*a*) Domain architecture of *Salpingoeca rosetta* synaptobrevin and *Homo sapiens* synaptobrevin 1 and 2. (*b*) Sequence alignment of the SNARE motif of *S. rosetta* synaptobrevin and *H. sapiens* synaptobrevin 1 and 2. The 15 layers (highlighted in blue including layers −1 to −7 and layers +1 to +8) important for SNARE complex formation are shown. The conserved arginine residues forming the ionic 0 layer are shown in green. (*c*–*c*″) Apical view of an *S. rosetta* cell stained with antibodies against (*c*) tubulin (grey) and (*c*′) synaptobrevin (yellow). (*c″*) Merged. (*d*–*d*″) Lateral view of a different *S. rosetta* cell stained with antibodies against (*d*) tubulin and (*d*′) synaptobrevin. (*d*″) Merged. The dashed square in (*d*″) indicates to position of (*h*). (*e*–*e*″) A rosette colony of *S. rosetta* stained with the same antibodies as in (*c*). The orange arrows indicate a basal synaptobrevin signal. (*e*) Tubulin. (*e′*) Synaptobrevin. (*e*″) Merged. The dotted square in (*e*″) indicates the position of (*f*). (*f*) Synaptobrevin-positive vesicles are in close contact with tubulin-positive cytoskeletal filaments. (*g*) TEM image showing the close contact between apical vesicles and tubulin filaments; av, apical vesicles; tf, tubulin filaments. (*h*) Image of a 3D reconstruction of the apical region of an *S. rosetta* cell. Apical vesicles are coloured in orange, tubulin filaments in light grey and the soma in half-transparent grey. Close contacts of vesicles and cytoskeletal filaments are indicated with white asterisks. The scale bar is 1 µm. *Sros, Salpingoeca rosetta*; *Hsap, Homo sapiens*.

The vesicle-associated SNARE protein synaptobrevin 1/2 (VAMP 1/2), together with Syntaxin 1 and SNAP-25 forms a stable complex that mediates the fusion of neurosecretory vesicles with the presynaptic plasma membrane. The formation of this so-called SNARE complex, which results in the release of neurotransmitters into the synaptic cleft, can be found in a variety of different animals [55]. Owing to its localization on neurosecretory vesicles and key role for vesicle exocytosis, we used synaptobrevin as a potential marker for putative secretory vesicles in the choanoflagellate *S. rosetta* for this study. The genome of the choanoflagellate *S. rosetta* encodes for a single synaptobrevin, which contains a highly conserved coiled-coil region responsible for SNARE complex formation [31,56] (figure 2*a*,*b*) and a single C-terminal transmembrane domain (figure 2*a*). *S. rosetta* synaptobrevin displays sequence identity to human synaptobrevin 1 of 38% and to human synaptobrevin 2 of 36%.

To assess the subcellular localization of synaptobrevin in *S. rosetta* by immunostaining, we raised a polyclonal antibody against the soluble portion of the protein (Syb [1-75]). To validate the specificity of the antibody, we performed western blot experiments. *S. rosetta* cell lysates probed with antibodies against synaptobrevin in the absence and presence of Syb [1-75] demonstrate that in the absence of Syb [1-75], the antibody recognizes a single band of approximately 11 kDa (electronic supplementary material, figure S1). Our immunohistochemical staining experiments revealed synaptobrevin localization predominantly to the apical, flagellum-bearing part of the cell (figure 2*c–f*). This confirms the previous results we obtained in *M. brevicollis* [57]. However, we also noted a weaker synaptobrevin signal localization at the basal part of the cell, suggesting the presence of secretory vesicles on two opposing sites of the cell. A very similar staining pattern was detected in *S. rosetta* cells of rosette colonies (figure 2*e*), supporting the finding detected in single cells. Additionally, we detected an overlap of the tubulin signal with single vesicles positive for synaptobrevin (figure 2*e*,*f*). This co-association of cytoskeletal (tubulin) filaments and at least apical vesicles is also present in transmission electron microscopy (TEM) sections revealed by 3D reconstruction (figure 2*g*,*h*). We did not detect synaptobrevin signals at putative plasma membrane contact sites, which are predominantly located in the median area of cell somata (figure 2*e″*).

### (c) Diverse and polarized vesicular landscape in choanoflagellates

**Figure 3.**
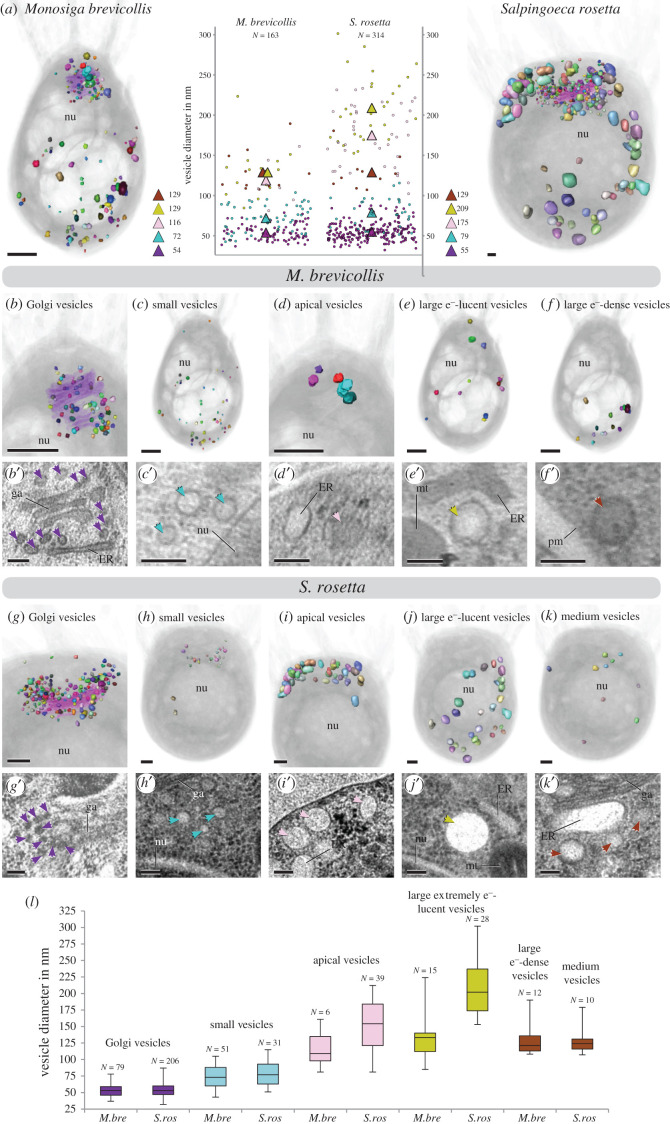
The diverse vesicular landscape of choanoflagellates. (*a*) Images of a 3D reconstruction of all vesicles in *M. brevicollis* (left) and *S. rosetta* (right). Individual vesicles are coloured randomly, and the cell is shown in half-transparent grey. A plot of all vesicle diameters measured is given in the middle. Mean diameters of different vesicle types are indicated by triangles in the same colours as in (*b–l*). (*b–f*) Visualization of separated vesicles of each vesicle type to show the localization within the soma of *M. brevicollis*. The Golgi-apparatus is shown in half-transparent lilac in (*b*). TEM images showing each vesicle type are given beneath images of the 3D model (*b′*–*f′*). (*g*–*k*) Visualization of separated vesicles of each vesicle type to show the localization within the soma of *S. rosetta*. The Golgi-apparatus is shown in half-transparent lilac in (*b*). TEM images showing each vesicle type are given beneath the 3D models (*g′*–*k′*). Scale bars of TEM images are 50 nm, scale bars of images of 3D reconstructions are approximately 250 nm. (*l*) Box and whiskers plots of the vesicle diameters within the different vesicle types (also see electronic supplementary material, table S2 and video S1 and S2). *M. brevicollis*: Golgi-associated vesicles (minimum: 37; median: 54; maximum: 79); small vesicles (minimum: 43; median 72; maximum 104); apical vesicles (minimum: 81; median: 109; maximum: 161); large extremely electron-lucent vesicles (minimum: 85; median: 132; maximum: 223); large electron-dense vesicles (minimum: 108; median 124; maximum 189). *S. rosetta*: Golgi-associated vesicles (minimum: 32; median: 55; maximum: 87); small vesicles (minimum: 51; median 78; maximum 116); apical vesicles (minimum: 102; median: 175; maximum: 233); large extremely electron-lucent vesicles (minimum: 153; median: 202; maximum: 301); medium vesicles (minimum: 107; median 125; maximum 180). *M.bre, Monosiga brevicollis*; *S.ros, Salpingoeca rosetta*. nu = nucleus, ga = Golgi apparatus, ER = endoplasmic reticulum, mt = mitochondria, pm = plasma membrane.

To investigate the number and diversity of vesicles in choanoflagellates we reconstructed the vesicular landscape in unicellular *M. brevicollis* (figure 3*a*, electronic supplementary material, video S1). In the specimen investigated we were able to identify 163 vesicles in total, which we assigned to five different gross vesicle types based on size, location and electron properties. (1) Electron-dense Golgi-associated vesicles (*N* = 79; mean diameter 54 nm), located in the apical region of the cell close to the Golgi apparatus (figure 3*b*,*b′*). In the reconstructed cell, a tubulus of the endoplasmic reticulum (ER) is located basally to the Golgi apparatus. Vesicles located between this ER tubulus and Golgi cisternae exhibit the same size as apical Golgi-associated vesicles but are more heterogeneous regarding their electron density (figure 3*b*′). However, they often appear slightly more electron-lucent compared to apical Golgi-associated vesicles, which could indicate a different cargo of these vesicles. (2) Small electron-lucent vesicles, resembling the vesicles between the Golgi-apparatus and ER tubulus but slightly larger (*N* = 51; mean diameter 72 nm), can be found in the whole cell body with a higher concentration in the basal area (figure 3*c*,*c*′). (3) Apical vesicles (*N* = 6; mean diameter 116 nm) are present in low numbers. They exhibit a higher electron density and are located in close proximity to the apical complex (figure 3*d*,*d′*). (4) Large extremely electron-lucent vesicles (*N* = 15; mean diameter 129 nm) are present in a scattered pattern within the whole cell soma (figure 3*e*,*e′*). (5) Large electron-dense vesicles (*N* = 12; mean diameter 129 nm) are present mainly in the basal third of the cell soma (figure 3*f*,*f′*).

To compare our findings with another choanoflagellate species we reconstructed the vesicular landscape of unicellular *S. rosetta* (figure 3*a*, electronic supplementary material, video S2). We were able to identify 314 vesicles in total, which we also assigned to five different gross vesicle types. (1) Electron-dense Golgi-associated vesicles (*N* = 206; mean diameter 55 nm) are located in the apical region of the cell close to the Golgi apparatus (figure 3*g*,*g′*). (2) Small electron-lucent vesicles (*N* = 31; mean diameter 79 nm) are found mainly in the apical region (figure 3*h*,*h′*) and resemble the Golgi-associated vesicles located between the basal Golgi apparatus and the ER tubulus in *M. brevicollis* (figure 3*b*,*b′*). However, the small vesicles in *S. rosetta* are very homogeneous regarding their electron density and therefore also resemble the small vesicles of *M. brevicollis* (figure 3*c*,*c′*). (3) Larger apical vesicles (*N* = 39; mean diameter 175 nm) are located in close proximity to the apical complex (figure 3*i*,*i′*). These vesicles are more electron-lucent and often more ovoid compared to the apical vesicles in *M. brevicollis*. (4) Large extremely electron-lucent vesicles (*N* = 28; mean diameter 209 nm) are present within the whole cell soma (figure 3*j*,*j′*). (5) A few medium-sized, electron-lucent vesicles (*N* = 10; mean diameter 129 nm) are present in a scattered pattern (figure 3*k*,*k′*).

In summary, we identified five distinct vesicle types for each of the two choanoflagellates species. While four vesicle types (e.g. Golgi-associated vesicles, small electron-lucent vesicles, apical vesicles, large extremely electron-lucent vesicles) are shared between the two species, large electron-dense vesicles are only present in *M. brevicollis* and medium vesicles are only present in *S. rosetta*.

### (d) Diversity of vesicles in two choanoflagellate species—commonalities and differences

A comparison between the vesicular landscapes of *M. brevicollis* and *S. rosetta* reveals differences in vesicle numbers of similar types, as well as of different vesicle types (figure 3*l*). Golgi-associated vesicles are of similar mean diameter in *S. rosetta* and *M. brevicollis*. However, *S. rosetta* exhibits 2.6 times more Golgi-associated vesicles than *M. brevicollis*. The mean diameter of small vesicles is also similar but slightly larger in *S. rosetta*. These vesicles differ in their abundance (1.6 times more in *M. brevicollis*) and cellular localization (compare figure 3*c*,*h*). Apical vesicles are different regarding their number (6.5 times more in *S. rosetta*), mean diameter (1.5 times larger in *S. rosetta*) and form (spherical in *M. brevicollis* and spherical to ovoid in *S. rosetta*). Similar differences can be observed for large (extremely) electron-lucent vesicles regarding their number (1.9 times more in *S. rosetta*) and mean diameter (1.6 times larger in *S. rosetta*). Large electron-dense vesicles of *M. brevicollis* show no similarities compared to the medium vesicles in *S. rosetta* and might represent different vesicle types.

## 3. Discussion

In agreement with previous studies [6,39,52,58,59], our results show that many components of the core proteome of neurosecretory vesicles have a pre-animal origin. In addition, we discovered that some of the vesicular transporters may be even older than previously thought owing to their presence in the fungus *B. dendrobatidis*. Furthermore, we showed the presence of a diverse vesicular landscape in choanoflagellates, the closest unicellular relatives of animals. Some of the vesicle types are closely associated with cytoskeletal components (tubulin filaments) and seem to be concentrated either apically or basally. This indicates the presence of a directed vesicular transport system in choanoflagellates, a characteristic shared by many neuronal cell types. In a variety of animal neuronal cell types, neurosecretory vesicles are concentrated either apically or basally and closely associated with tubulin filaments [60]. The observed similarities in vesicular protein composition and landscape in choanoflagellates and animal nerve cells could be explained by the following hypothesis:

The components of neurosecretory vesicles were present in the last common ancestor of holozoans. There they might have had a more general function, such as the secretion of substances for the extracellular matrix (ECM) or enzymes, additional to cell–cell signalling. Despite the already established role in cell–cell communication, signalling molecules were not transmitted directly to neighbouring cells but rather secreted into the surrounding environment. During the evolution of animals this pre-existing signalling machinery has been moved to cell–cell contact sites leading to a more efficient and specific signal transduction, as can be seen in synapses of recent animals. In this scenario, the function (secretion of signalling molecules) and the biological role (cell–cell signalling) would be ancestral, while the localization at cell-membrane contact sites would represent an evolutionary novelty [61].

Our 3D reconstructions of two unicellular choanoflagellate cells revealed a diverse vesicular landscape. Vesicles are highly dynamic organelles, so we acknowledge that it can be problematic to assign single vesicles to one of the gross types identified, since they exhibit intermediate features (diameter, electron density, localization) between types. The large whiskers in figure 3*l* are a visualization of this problem. However, since either the median values or electron densities (or both) of the assigned vesicle types are very different from each other, our defined vesicle types are highly likely to be real. In the choanoflagellates *S. rosetta* and *M. brevicollis*, we identified morphologically distinct vesicle populations at both the basal and apical poles of the cell.

Both vesicle populations are potentially secretory, as we found that the vesicle-associated SNARE protein synaptobrevin is localized to the apical and basal parts of *S. rosetta.* We can only speculate about the content and function of these vesicles. Vesicles localized to the basal pole of *S. rosetta* could potentially contain the C-type lectin Rosetteless [62], or other extracellular matrix material [63], as their basal secretion seems to be essential for multicellular rosette development [63]. Vesicles at the apical pole, close to the feeding collar in choanoflagellates, might contain mucus and digestive enzymes for external digestion [64]. Alternatively, these vesicles could also contain sialic acid, aspartate or glutamate [8], which might serve for communication between cells, as our comparative analysis revealed putative sialin-like transporters [65] in the genomes of both *M. brevicollis* and *S. rosetta* (figure 1 and electronic supplementary material, table S1).

**Figure 4.**
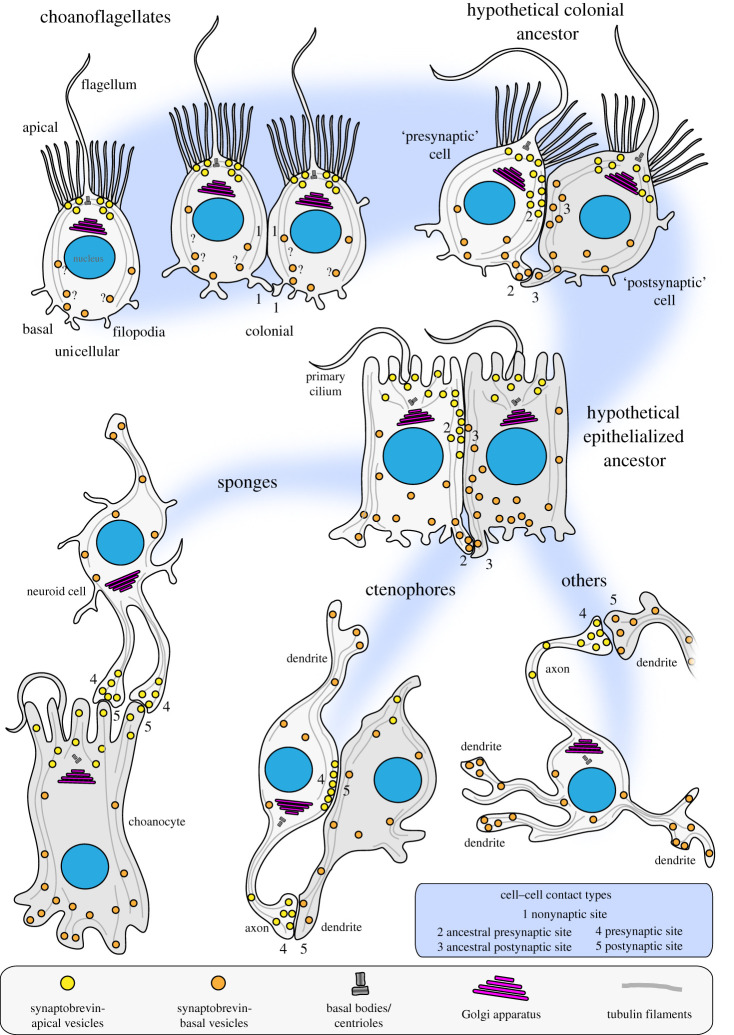
An evolutionary scenario for the structural evolution of animal synapses. A polarized vesicle transport system might have already existed in choanoflagellates. No chemical signal transduction appears at soma or filopodial plasma membrane contact sites (1). In the stem lineage of animals, the apical–basal directed vesicle transport has been translocated to soma and/or filopodial plasma membrane contact sites. This resulted in one cell acting as a signal donor or ancestral presynapse (2) and another cell acting as a signal receiver or ancestral postsynapse (3). This relationship might have been further stabilized in a more epithelialized, obligate multicellular animal ancestor. From this condition the evolution of more stable presynaptic (4) and postsynaptic (5) relationship was achieved in different groups of early branching animals.

Despite the lack of data on vesicular cargo and the function of vesicular signalling molecules in unicellular holozoans we propose a choanoflagellate-biased scenario for the structural evolution of neurosecretory cell–cell signalling (figure 4). Most of the structural components of neurosecretory vesicles, including a polarized apical-to-basal vesicular transport system, were present in the last common ancestor of choanoflagellates and animals. In colonial choanoflagellates, plasma membrane contacts between cells are present but not involved in chemical cell–cell signalling, indicated by a lack of vesicles at the cellular contact sites. However, they might be involved in the exchange of other biomolecules that are not stored in vesicles, or in electrical signalling. In the stem lineage of animals, neurosecretory vesicles were recruited to plasma membrane contact sites (soma or filopodial contacts) and used for intracolonial communication. This resulted in the emergence of a ‘presynaptic’ (signal donor) and ‘postsynaptic’ (signal receptor) cell. This condition might have been further stabilized in the ‘epithelialized’ last common ancestor of animals. From this condition many different structural types of synapses might have evolved such as, for example, the neuroid–choanocyte relationship in a sponge [66], pre-synaptic triads and somatic synapses in ctenophores [67] and ‘classical’ synapses present in most other animals. In this scenario, the structural co-option of ancestral neurosecretory vesicles and polarized vesicular transport at plasma membrane contact sites might be the key process leading to the structural evolution of animal synapses. However, more studies on the presence, intracellular localization and function of classical ‘animal’ neurotransmitters in unicellular holozoans are needed to elucidate the ancestral function of the neurosecretory vesicle machinery.

## 4. Methods

### (a) Protein searches and analysis

Putative protein homologues of neurosecretory vesicle proteins were found using the basic local alignment sequence similarity search tool (BLASTp) [68] at the National Center for Biotechnology Information (NCBI), National Human Genome Research Institute (NIH) and EnsemblMetazoa database. As queries we used protein sequences from *Homo sapiens* and *Rattus norvegicus* for the detection of neurosecretory vesicle proteins in seven other animals (*D. rerio*, *S. purpuratus*, *D. melanogaster*, *N. vectensis*, *T. adhaerens*, *M. leidyi* and *A. queenslandica*), three protists (two choanoflagellates (*M. brevicollis* and *S. rosetta*) and one filasterean (*C. owczarzaki*) and three fungi (*B. dendrobatidis*, *R. oryzae*, *S. cerevisiae*). We performed protein homology-based searches with the default BLAST parameters and an e-value threshold of less than or equal to 1 × 10^−6^. Initially obtained sequences were reciprocally searched against the NCBI protein database using BLASTp to verify results. Protein sequences of putative homologues were further analysed using the protein domain prediction programs Pfam [69] and SMART [70,71] to demonstrate the conservation of protein domain families and domain organizations/arrangements. Protein accession numbers, domain composition and time points of the investigations are shown in the electronic supplementary material, table S1.

### (b) *Salpingoeca rosetta* and *Monosiga brevicollis* cell culture

Colony-free *S. rosetta* cultures (50818, American Type Culture Collection) were grown with co-isolated prey bacteria in 0.22 µm filtered choanoflagellate growth medium diluted at a ratio of 1 : 4 with autoclaved seawater as previously described [42]. Cultures were maintained at 18°C and split 1.5 : 10 once a week.

*M. brevicollis* cultures (50154; American Type Culture Collection) were cultured in artificial seawater mixed with Wards cereal grass medium in a 1 : 1 ratio, adjusted to a salt concentration of 53 mS cm^−1^ and sterile filtered as previously described [57]. Cultures were maintained at 25°C and diluted 1 : 100 once a week.

### (c) Protein expression and purification of *S. rosetta* synaptobrevin

To express and purify *S. rosetta* synaptobrevin a codon-optimized nucleotide sequence encoding the soluble portion of the protein [Syb (1-75)] was prepared by gene synthesis (Genscript, USA): GAGGCGAACCGTACCGGTGACTACCGTCTGCAGGAAGCGCAGCGTCAAGTGGGCGAAGTTCAAAACGTGATGCGTGATAACCTGACCAAGGTTATCGAGCGTGGTGAAAAACTGGACGATCTGGACGCGAAGGCGGAAGATCTGGAGGCGGAGGGTCAGCGTTTCCAAAACCGTGCGGGCCGTCTGCGTCGTCAGATGTGGTGGCAAAACAAACGTAACCAGTAA

This sequence was cloned into a pET28a(+) vector (69864, Novagen), which contains an N-terminal, thrombin-cleavable His6-tag. *Escherichia coli* BL21(DE3) Singles Competent Cells (70235, Novagen) were subsequently transformed. Following this, [Syb (1-75)] was expressed at 37°C for 3 h and purified by Ni^2+^-nitrilotriacetic acid (NTA) chromatography. For this, *E. coli* cells were pelleted at 3488× g for 10 min at 4°C (Heraeus Megafuge 40R) and incubated at room temperature for 20 min with 100 µl of 200 mM phenylmethanesulfonyl fluoride (PMSF) (36978, ThermoFisher Scientific) and lysozyme from chicken egg white (L6876, Sigma-Aldrich). Following incubation, cells were sonicated (Vibra-CellTM, Sonics) by 3 × 30 s pulses and incubated for 10 min at room temperature with 100 µl of 1 M MgCl_2_, 500 µl of 20% Triton X-100 and Deoxyribonuclease 1 from bovine pancreas (DN25, Sigma-Aldrich). Cellular debris was removed by centrifugation at 5488× g for 40 min at 4°C (Heraeus Megafuge 40R) and incubated for 2 h with 500 µl HisPurTM Cobalt Resin (89964, ThermoFisher Scientific) at 4°C. The beads were then pelleted at 1363× g for 10 min at 4°C, the supernatant was removed, and the beads washed three times in wash buffer (500 mM NaCl, 20 mM Tris pH 7.4). His-tagged proteins were eluted from the beads in a disposable polypropylene column (29924, ThermoFisher Scientific) using elution buffer (4 ml wash buffer containing 400 mM imidazole) and dialyzed overnight in Biodesign Cellulose Dialysis 3.5 kDa tubing (12757496, ThermoFisher Scientific) at 4°C in dialysis buffer (100 mM NaCl, 20 mM Tris pH 7.4, containing 15 µl bovine thrombin (605157, Merck Millipore) to cleave the His-tags). Protein eluates were further purified by ion exchange chromatography using an Äkta Prime Plus equipped with a HiTrapTM SP HP column (GE Healthcare, Sweden) and eluted along a linear gradient of NaCl in 20 mM Tris, pH 7.4, 1 mM EDTA. The success of the purification was assessed by sodium dodecyl sulfate polyacrylamide gel electrophoresis (SDS-PAGE) on a 16% gel, run using an XCell SureLock MiniCell chamber (ThermoFisher Scientific, USA) and stained with SimplyBlue SafeStain (LC6060, ThermoFisher Scientific). Protein concentrations were quantified by measuring absorbance at 280 nm using a NanoDrop 1000 Spectrophotometer (ThermoFisher Scientific, USA).

### (d) *Salpingoeca rosetta* synaptobrevin antibody production

Polyclonal antibodies were commercially raised in rabbits against recombinant [Syb (1-75)] antigen (Covalab, UK). Immunoglobulins were purified against Protein A from 1 ml of rabbit serum using a Protein A HP SpinTrap (28-9031-32, GE Healthcare) following the manufacturer's instructions.

### (e) Western blot analysis

To assess the specificity of the anti-synaptobrevin antibody a competitive indirect western blot analysis was performed (electronic supplementary material, figure S1). A 30 mg wet weight *S. rosetta* cell pellet was resuspended in 1.6 ml lysis buffer (20 mM potassium phosphate buffer, pH 7.4, 150 mM NaCl, 1 mM EDTA, 1 mM ethyleneglycoltetraacetic acid [EGTA], 1% Triton X-100) containing protease inhibitor cocktail (Roche) and centrifuged for 10 min at 4°C and 13 000× g. The lysate (225 or 450 µg wet weight) was loaded on a gradient sodium dodecyl sulfatepolyacrylamide gel (SDS-PAGE) and electrophoresis was performed at room temperature, at 100 mV. The separated proteins were blotted onto a 0.2 µm PVDF membrane, blocked for 1 h at room temperature on a shaker in blocking buffer (5% non-fat milk in PBS containing 0.1% Tween 20) and cut into pieces prior to immunostaining at 4°C overnight on a shaker. Before immunostaining, the anti-synaptobrevin antibody was preabsorbed with different concentrations of recombinant synaptobrevin protein (4 and 40 µg) and 40 µg BSA in blocking buffer (1 : 1000) at room temperature for 30 min. On the next day, the membrane was washed extensively in PBS-T and stained with secondary antibody goat anti-rabbit IgG, horseradish peroxidase conjugate (ab97051; abcam) in blocking buffer (1 : 10 000) for 1 h at room temperature. The membrane was washed again in PBS-T and the staining was visualized using the Clarity Max Western ECL Substrate (BioRad).

### (f) Immunofluorescence microscopy

Prior to fixation, cells were pelleted by gentle centrifugation (500× g for 10 min at 4°C) in a Heraeus Megafuge 40R (ThermoFisher Scientific) and resuspended in a small volume of culture medium. Concentrated cell suspension (500 µl) was applied to glass-bottom dishes coated with poly-l-lysine solution (P8920, Sigma-Aldrich) and left for 10–30 min until cells were sufficiently adhered. Cells were fixed in 200 µl of ice-cold 6% acetone in 4× PBS for 5 min and then 4% paraformaldehyde in 4× PBS for 15 min. Fixing solutions were then aspirated off, washed twice in 4× PBS, twice in 2× PBS and once in 1× PBS and then blocked for 30 min in blocking buffer (1% bovine serum albumin (BSA) and 0.6% Triton X-100 in PEM solution (100 mM piperazine-N,N′-bis(2-ethanesulfonic acid) (PIPES) at pH 6.9, 1 mM EGTA, and 0.1 mM MgSO4)). Cells were then incubated with primary antibodies (mouse monoclonal antibody against β-tubulin (E7, 1 : 200; Developmental Studies Hybridoma Bank, USA) and anti-[Syb(1-75)] antibody, 1 : 500) in 200 µl of blocking buffer for 1 h, washed 4 times in blocking buffer and then incubated in the dark for 1 h with secondary antibodies in 200 µl of blocking buffer (polyclonal goat anti-mouse Alexa Fluor 488, 1 : 200 (A32723, ThermoFisher) and goat anti-rabbit Alexa Fluor 647, 1 : 200 (A-21244, ThermoFisher)). Dishes were then washed four times in blocking buffer, washed once in 1× PBS and finally mounted under coverslips with ProLong Gold Antifade Mountant (P36935, ThermoFisher Scientific). Single choanoflagellate cells were imaged using a Zeiss LSM 510 confocal microscope. Colonial choanoflagellate cells were imaged using a Zeiss Axio Observer LSM 880 with an Airyscan detector.

### (g) Transmission electron microscopy (TEM)

TEM of *S. rosetta* cells was performed essentially as described [42]. In brief, *S. rosetta* cells were high-pressure frozen in a Bal-Tec HPM 010 high-pressure freezer (Bal-Tec AG, Liechtenstein). Freeze substitution with 1% osmium tetroxide plus 0.1% uranyl acetate in acetone was performed over 2 h by the SQFS method of McDonald & Webb [72], then infiltrated with Eponate 12 resin and polymerized in a Pelco Biowave research microwave oven (Ted Pella, Inc., Redding, CA) over a period of 2 h. Sections were cut at 70 nm thickness, poststained with uranyl acetate and lead citrate, and viewed in a Tecnai 12 transmission EM (FEI Inc., Hillsboro, OR).

TEM of *M. brevicollis* cells was performed essentially as described [57]. In brief, for *M. brevicollis* electron microscopy, cells were flash-frozen in a Baltec HPM 010 high-pressure freezer. Cryosubstitution and embedding were performed in a Leica EM AFS. Cells were sequentially incubated at low temperature (−90°C) in 0.1% tannic acid (100 h) and 2% OsO_4_ (7 h) in acetone. They were progressively brought to room temperature before being embedded in Epon (Electron Microscopy Sciences) and polymerized 24 h at 60°C. Ultrathin 70 nm sections were cut and contrasted with uranyl acetate and lead citrate before being observed in a LEO 912 AB (Zeiss).

### (h) 3D Reconstruction and analysis

To better recognize thin membranous outlines of vesicles, contrast was enhanced using the CLAHE plugin in Fiji [73,74] for the image stack of *S. rosetta* prior to the reconstruction. Digital image stacks of the TEM sections of *M. brevicollis* and *S. rosetta* were imported into AMIRA (FEI Visualization Sciences Group) and aligned semi-manually. Subsequently, single vesicles were segmented manually by tracing structures along the *z*-axis and 3D reconstructed by automatically merging the traced parts. In some cases, there were fluent transitions between large vesicles and isolated smaller parts of the smooth endoplasmic reticulum. For consistent results, membranous structures were defined as vesicles when they could be traced over a maximum of three sections and as part of the smooth endoplasmic reticulum when they were larger than three sections. For surface reconstructions, single surface models for each vesicle were rendered from the segmented materials, numbers of vertices were reduced around ten times and the surfaces were smoothened. The cell soma, collar and flagellum were visualized and merged with the vesicle surface models using the volume rendering function in AMIRA. Vesicle diameters were calculated using the 3D measuring tool in AMIRA. For every vesicle, the largest distance between two points on the vesicular membrane, evaluated by eye, was measured. If vesicles extended over several (maximum three) sections, the diameter was measured on the section with the largest surface area. All measurements were conducted using unprocessed, unsmoothed materials. Subsequently, all measurements were exported to Microsoft Excel 2010 (Microsoft Corporation) to prepare point graphs and box and whisker plots.

## Data accessibility

The datasets supporting this article have been uploaded as part of the electronic supplementary material.
